# The impact of doubling dairy or plant-based foods on consumption of nutrients of concern and proper bone health for adolescent females

**DOI:** 10.1017/S1368980016002901

**Published:** 2016-11-10

**Authors:** Elieke Demmer, Christopher J Cifelli, Jenny A Houchins, Victor L Fulgoni

**Affiliations:** 1 National Dairy Council, 10255 West Higgins Road, Suite 900, Rosemont, IL 60018-5616, USA; 2 Nutrition Impact, LLC, Battle Creek, MI, USA

**Keywords:** Female adolescents, Diet modelling, Nutrients of concern, Plant-based diet, Dairy

## Abstract

**Objective:**

To determine the effects of increasing plant-based foods *v*. dairy foods on energy and nutrients of concern in adolescent females via diet modelling exercises.

**Design:**

Data from the National Health and Nutrition Examination Survey (NHANES) were used to compare nutrient intakes from usual diet with those from three dietary scenarios that increased current intakes by 100 % of the following: (i) plant-based foods; (ii) protein-rich plant-based foods; and (iii) milk, cheese and yoghurt. The first two scenarios had commensurate reductions in animal products.

**Setting:**

What We Eat in America, NHANES 2007–2010.

**Subjects:**

Female adolescents (*n* 1594) aged 9–18 years.

**Results:**

When currently consumed plant-based foods were increased by 100 %, there were increases in dietary fibre, added sugar, vitamin E, Fe and folate intakes. These increases were accompanied by decreases in total fat, saturated fat, Zn, vitamin D, Ca and protein intakes. Protein-rich plant foods are consumed in very low quantities in this population such that doubling their intake resulted in no real nutritional impact. When dairy products were increased by 100 % there were increases in intakes of vitamin D, Mg, Zn, Ca, K, energy, saturated fat and protein.

**Conclusions:**

Non-specific recommendations to increase plant foods can lead to unintended nutritional consequences. For adolescent girls, meeting the dietary recommendation of three daily servings of dairy improved the intake of the identified nutrients of concern while simultaneously providing adequate nutrients essential for proper growth and bone health critical during the adolescent phase.

Adolescence is characterized by accelerated growth, second only to infancy^(^ ^1^ ^)^. This growth spurt increases the need for several nutrients, including vitamin D, Fe, Zn, folate, energy and protein^(^ ^2^ ^,^ ^3^ ^)^. Additionally, many diet and lifestyle habits acquired during adolescence carry over into adulthood and can last a lifetime^(^ ^4^ ^)^. Hence, this period is an important window of opportunity to significantly impact lifelong health.

While both adolescent boys and girls are nutritionally vulnerable, adolescent girls generally fare the worst in not consuming adequate nutrients. Lifestyle choices involving dieting and meal skipping due to body image issues and the desire to be thin are contributing factors^(^ ^4^ ^)^. Adolescent girls are less likely to consume three meals per day and less likely to eat breakfast than their male counterparts^(^ ^5^ ^,^ ^6^ ^)^. Thus, it is crucial that the foods this population consume be nutrient-rich, such as milk, cheese and yoghurt. Milk is a good or excellent source of protein, Ca, vitamin A, vitamin D, vitamin B_12_, riboflavin, niacin, P and pantothenic acid^(^ ^7^ ^)^. In addition, some studies have shown that milk and milk product consumption during adolescence is associated with neutral or reduced risk of adiposity^(^ ^8^ ^–^ ^12^ ^)^ and reduced blood pressure with either higher intake^(^ ^13^ ^)^ or as part of a DASH (Dietary Approaches to Stop Hypertension)-type dietary pattern^(^ ^14^ ^)^, and some studies link consumption of milk and milk products to dental health^(^ ^15^ ^)^. However, current estimates of total dairy intakes by adolescent girls are well below the 2015 Dietary Guidelines for Americans’ recommendation of 3 servings/d^(^ ^6^ ^)^ as well as the American Academy of Pediatrics’ recommendation of 4 dairy servings/d^(^ ^16^ ^)^.

The 2015 Dietary Guidelines for Americans report that vitamin D, Ca, P, fibre and Fe are nutrients of concern for adolescent females^(^ ^6^ ^)^. Based on data from the third National Health and Nutrition Examination Survey (NHANES III), the prevalence of Fe deficiency in US girls aged 12–19 years was between 8 and 10 % of the population. During adolescence there is an increase in dietary Fe requirement due to the expansion of total blood volume associated with growth, and in adolescent girls this requirement is greater to account for the amount of Fe lost in menses^(^ ^17^ ^)^. The National Growth and Health Study evaluated the longitudinal food intake of 2379 girls during the periods of 9–13, 14–18 and 19–20 years of age and found at each age group that more than 90 % did not consume the recommended amounts of fruit, vegetables or dairy^(^ ^18^ ^)^. Inadequate intakes of Ca, Mg, K and vitamins D and E were found in the majority of girls in all age groups. Generally, as the girls aged micronutrient inadequacy worsened.

Recently, there has been increased discussion around the role of animal *v*. plant foods and their associated environmental impacts (e.g. impact on greenhouse gas emissions or land use) to ensure that current and future generations are able to meet their dietary needs for a healthy lifestyle. A plant-centred diet has been shown to lower the risk of diet-related chronic disease^(^ ^6^ ^,^ ^19^ ^–^ ^21^ ^)^ and has been suggested to be more environmentally sustainable^(^ ^22^ ^–^ ^24^ ^)^. Furthermore, a key message from the Institute of Medicine workshop on healthy sustainable diets stated that plant-based protein sources are worth considering as an alternative to animal-based protein sources^(^ ^25^ ^)^. However, it is unclear what the nutritional impact will be on adolescent females if they attempt to reduce the consumption of animal products and increase plant-based foods in their diet. Hence, the objective of the present study was to determine the effects of increasing plant-based foods *v*. increasing dairy foods on energy and nutrient adequacy in adolescent girls. Diet modelling exercises were conducted based on the following scenarios: (i) increasing the amount of plant-based foods by 100 % based on current consumption patterns; (ii) increasing the amount of protein-rich plant foods (i.e. legumes, nuts, seeds, soya) that are currently consumed by 100 %; and (iii) increasing the amount of dairy foods currently consumed by 100 %. Our hypothesis was that doubling foods that are already consumed, in specific food groups, would be easier to achieve than imposing a behaviour change that required increasing the intakes of foods not currently consumed by adolescents.

## Methods

Data from What We Eat in America, the dietary component of the NHANES 2007–2010, were used in all of our analyses. NHANES is a nationally representative cross-sectional study that utilizes a stratified, multistage probability sample of the non-institutionalized US population^(^ ^26^ ^)^. Food and nutrient intakes were determined from two non-consecutive 24 h dietary recalls. The first was conducted by an in-person interview at the mobile examination centre and the second was obtained via a telephone interview a few days later. Only data coded as reliable by the US Department of Agriculture for adolescent females aged 9–18 years were used (*n* 1594). The National Cancer Institute method was used for estimating usual intakes and the covariates used were day sequence, Dietary Reference Intake age groups and recall weekday/weekend indicators^(^ ^27^ ^)^.

Three dietary scenarios were examined in the present study and compared with the usual diet. To model what could happen if adolescent females followed a blanket recommendation of increasing plant-based foods at the expense of animal protein, scenario 1 increased the currently consumed plant-based foods (fruits, vegetables, total grains, legumes, nuts, seeds and soya) by 100 % while animal protein intake (eggs, meat, poultry, fish and dairy) was proportionately decreased based on usual consumption on a gram-per-gram basis. This approach was undertaken because dietary habits are typically difficult to change and it may be easier for individuals to double the plant-based foods they currently consume instead of introducing new plant-based foods. Since it was anticipated that overall protein intake would decrease when currently consumed plant-based foods were increased and animal products were reduced (scenario 1), scenario 2 modelled the impact of increasing currently consumed protein-rich plant-based foods (i.e. beans, peas, legumes, nuts, seeds and soya products) by 100 % with commensurate decreases in animal products on a gram-per-gram basis.

For scenario 3, currently consumed dairy foods (milk, cheese and yoghurt) were increased by 100 %. Dietary guidelines in the USA and elsewhere recommend adolescents consume 3–4 servings dairy products/d for optimal nutrition^(^ ^19^ ^,^ ^28^ ^,^ ^29^ ^)^; therefore, scenario 3 modelled the impact of meeting these dietary recommendations by doubling the dairy foods currently consumed without any further adjustments to the diet. This scenario used the US Department of Agriculture’s dairy composite nutrient profile for the increased dairy products.

Mixed dishes containing dairy products (e.g. macaroni and cheese), plant foods (e.g. bean soup) or eggs, meat, poultry and fish were not increased or decreased in any of the scenarios due to the difficulty associated with accurately disaggregating NHANES data to identify which nutrients are coming from which ingredients in these mixed dishes. Only those foods eaten as consumed were increased or decreased in the analysis presented herein, which allowed for a direct comparison between changes in food intake and nutrient adequacy.

The Food Pattern Equivalent Database for each NHANES release was used to define various types of foods in the analyses^(^ ^30^ ^,^ ^31^ ^)^. Plant-based foods were defined as food codes with zero servings of dairy, meat/poultry/fish and eggs with non-zero servings for the sum of servings of fruits, vegetables, legumes, grains, soya products and nuts/seeds. Animal-based foods were defined as food codes with zero servings for the sum of servings of fruits, vegetables, legumes, grains, soya products and nuts/seeds with non-zero servings for the sum of dairy, meat/poultry/fish and eggs. Higher protein-based foods were defined using the US Department of Agriculture’s food category ‘Plant-based Protein Foods’. This category includes ‘Beans, peas and legumes’, ‘Nuts and seeds’ and ‘Processed soy products’^(^ ^32^ ^)^. Nutrient composition of dairy was based on the dairy food composite used by the US Department of Agriculture for dietary pattern analyses^(^ ^33^ ^)^. Adequate protein intake was based on grams of protein per kilogram of body weight and grams of protein per kilogram of ideal body weight as recommend by the Dietary Reference Intakes^(^ ^34^ ^)^.

The statistical software packages SAS 9.2 and SUDAAN 11 were used for all analyses. NHANES survey weights strata and primary sampling units were also used in all calculations. The National Cancer Institute method was used to estimate usual intakes of the food groups and selected nutrients to assess the percentage of the population meeting nutrient adequacy^(^ ^25^ ^,^ ^27^ ^)^. Given that we know there are differences in modelling scenarios by design, typical statistical testing was not appropriate. Thus, to assess meaningful differences in changes of the means for the modelling scenarios we examined means and their 95th percentile confidence limits. Non-overlapping confidence intervals we deemed meaningful. This approach has been utilized previously in dietary pattern studies^(^ ^35^ ^)^.

## Results

Table 1 lists the food groups usually consumed by adolescent females prior to dietary modelling. Grain-based foods comprised the largest food group, followed by the meat, poultry and fish group and the dairy group. The least consumed food groups were legumes, soya, and nuts and seeds. Due to the low intakes of foods in these categories, the corresponding decrease in animal-based foods was lower in scenario 2 (where currently consumed protein-rich plant-based foods, such as legumes, nuts and seeds, and soya products, were increased by 100 %) compared with scenario 1 (where all plant-based foods were increased by 100 %). Based on their usual intake, adolescent girls consumed 1·8 cup-equivalents dairy/d. In scenario 3, currently consumed milk, cheese and yoghurt was increased by 100 %, increasing the total dairy to approximately 3 cup-equivalents/d, meeting the recommendation of the 2015–2020 Dietary Guidelines for Americans^(^ ^36^ ^)^. Changes in individual food group intakes were based on each food as consumed and did not account for those foods in mixed dishes. As such, there was a range of increases and decreases, which reflects differences in how each food is consumed.

**Table 1:** Usual daily food group intakes prior to dietary modelling in adolescent females aged 9–18 years, National Health and Nutrition Examination Survey (NHANES) 2007–2010

	Usual diet	
Food group	Mean	se
Eggs (ounce-equivalents)	0·35	0·03
Legumes (cup-equivalents)	0·07	0·01
Meat, poultry, fish (ounce-equivalents)	3·22	0·11
Nuts and seeds (ounce-equivalents)	0·35	0·04
Soya (ounce-equivalents)	0·04	0·01
Total dairy (cup-equivalents)	1·78	0·05
Total fruit (cup-equivalents)	0·90	0·06
Total grains (ounce-equivalents)	6·57	0·22
Total vegetables (cup-equivalents)	0·96	0·03

Tables 2 and 3 show the usual consumption of macronutrients, the nutrients identified to be of concern for adolescent females and the shifts observed with each of the three dietary modelling exercises. A 100 % increase in all plant foods currently consumed (scenario 1) resulted in decreased intakes of protein, total fat and saturated fat and increased intakes of carbohydrate, dietary fibre and added sugars compared with the usual diet. Protein intake was lower by almost 10 g/d in scenario 1 when compared with the usual intake (Table 2) and the proportion of adolescent females not meeting the Estimated Average Requirement (EAR) for protein almost tripled in scenario 1 (Table 3). Total fat and saturated fat each decreased by about 4 g/d. Dietary fibre increased by about 4 g/d, increasing the proportion of adolescent females who had intakes above the Adequate Intake (AI) by 8 %. Added sugar intake increased by about 1 g/d. Increased consumption of plant foods also impacted vitamin D, vitamin E, folate, Zn and Ca intakes. There was an improvement, or a decrease, between the usual diet and scenario 1 for the percentage of adolescent females not meeting the EAR for vitamin E and folate (Table 3). However, the percentage of adolescent females not meeting the EAR increased for Ca, Zn and vitamin D between the usual diet and scenario 1. Fe intake increased but the percentage not meeting the EAR did not significantly differ from usual intakes.

**Table 2:** Daily macronutrient intakes from the usual diet compared with a 100 % increase in plant foods, a 100 % increase in protein-rich plant foods and a 100 % increase in dairy foods in adolescent females aged 9–18 years, National Health and Nutrition Examination Survey (NHANES) 2007–2010*

	Usual diet		100 % Increase in all plant foods (scenario 1)		100 % Increase in protein-rich plant foods (scenario 2)		100 % Increase in dairy foods (scenario 3)	
Nutrient	Mean	se	Mean	se	Mean	se	Mean	se
Energy (kJ)	7626·2	97·1	7936·2	120·1	7678·9	99·6	8507·3†	109·6
Energy (kcal)	1822·7	23·2	1896·8	28·7	1835·3	23·8	2033·3†	26·2
Carbohydrate (g)	241·8	3·6	284·0†	3·9	243·4	3·5	243·4	4·1
% Energy from carbohydrate	52·9	0·3	59·7†	0·4	52·8	0·4	51·7	0·3
Protein (g)	63·7	0·9	54·0†	1·0	64·2	1·0	74·5†	1·1
% Energy from protein	14·1	0·2	11·3†	0·1	14·1	0·2	14·7†	0·2
Total fat (g)	68·0	1·0	63·5†	1·4	69·6	1·1	77·8†	1·1
% Energy from fat	32·6	0·3	28·7†	0·4	32·8	0·3	33·3	0·3
SFA (g)	23·4	0·4	19·0†	0·5	23·4	0·4	29·3†	0·4
% Energy from SFA	11·2	0·1	8·7†	0·1	11·1	0·1	12·4†	0·1
Dietary fibre (g)	13·0	0·3	16·9†	0·4	13·5	0·3	13·5	0·3
Added sugars (teaspoon-equivalents)	19·2	0·4	20·2†	0·4	19·2	0·4	21·2†	0·4

* Each of the modelling exercises was based on plant-based foods, protein-rich plant-based foods or dairy foods as consumed. The models increased/decreased foods as consumed and did not change the individual foods found in mixed dishes.

† Meaningfully different from baseline due to non-overlapping 95th percentile confidence interval.

**Table 3:** Percentage of adolescent females (9–18 years) with daily nutrient intakes below the Estimated Average Requirement (EAR) or above the Adequate Intake (AI) based on usual intake, a 100 % increase in plant foods, a 100 % increase in protein-rich plant foods and a 100 % increase in dairy foods, National Health and Nutrition Examination Survey (NHANES) 2007–2010*

	Usual diet				100 % Increase in plant foods (scenario 1)				100 % Increase in protein-rich plant foods (scenario 2)				100 % Increase in dairy foods (scenario 3)			
Nutrient	UI	se	% <EAR	se	UI	se	% <EAR	se	UI	se	% <EAR	se	UI	se	% <EAR	se
EAR																
Vitamin D (μg)	4·5	0·2	96·9	0·7	2·9†	0·2	99·7	0·2	4·4	0·2	97·4	0·6	7·0†	0·3	79·6†	1·8
Vitamin E (mg)	6·1	0·1	93·5	0·6	7·2†	0·1	84·9†	1·1	6·5	0·1	89·8†	0·8	6·3	0·1	92·2	0·8
Folate (μg)	496·4	10·7	11·2	1·3	660·1†	15·1	4·8†	0·9	504·5	10·4	10·5	1·3	510·1	11·0	10·0	1·3
Mg (mg)	220·9	4·1	65·2	1·9	236·1	4·5	58·9	1·9	227·8	4·1	62·5	1·9	255·8†	4·9	51·2†	2·2
Zn (mg)	9·4	0·2	21·5	2·4	8·9	0·2	33·2†	2·6	9·6	0·2	20·7	2·3	10·9†	0·2	12·1†	1·8
Fe (mg)	13·2	0·2	5·1	0·7	16·6†	0·4	3·0	0·6	13·4	0·3	4·9	0·7	13·6	0·3	4·4	0·7
Ca (mg)	904·4	17·9	75·9	1·9	726·3†	13·9	92·1†	1·1	894·4	18·0	77·1	1·8	1278·1†	29·7	41·3†	2·4
Protein (g/kg BW)	1·2	0·0	5·4	1·0	1·1†	0·0	14·5†	2·0	1·3	0·0	5·4	1·1	1·5†	0·0	2·8	0·7
	UI	se	% >AI	se	UI	se	% >AI	se	UI	se	% >AI	se	UI	se	% >AI	se
AI																
Dietary fibre (g)	13·0	0·3	1·3	0·3	16·9†	0·4	9·5†	1·1	13·5	0·3	2·3	0·5	13·5	0·3	1·7	0·4
K (mg)	1977·3	30·5	0·0	0·0	2006·1	30·5	0·0	0·0	2004·8	30·3	0·0	0·0	2367·2†	36·6	0·8†	0·2
Na (mg)	2981·5	51·7	99·3	0·3	3013·9	64·5	98·9	0·4	2987·5	52·1	99·2	0·3	3231·5†	51·2	99·6	0·2

UI, usual intake; BW, body weight.

* Each of the modelling exercises was based on a 100 % increase in plant-based foods, protein-rich plant-based foods or dairy foods as consumed. The models increased/decreased foods as consumed and did not change the individual foods found in mixed dishes.

† Meaningfully different from baseline due to non-overlapping 95th percentile confidence intervals.

In scenario 2, which increased the consumption of protein-rich plant foods by 100 %, there were no meaningful changes in macronutrients or the specific micronutrients examined compared with usual intakes. One exception was vitamin E intake, which showed an improvement for the percentage of girls not meeting the EAR via a decrease of 3·7 % (Table 3).

A 100 % increase in currently consumed dairy (scenario 3) led to an increase in energy, protein, total fat, saturated fat and added sugars, compared with usual intake levels. Protein consumption rose by 10·8 g/d and the percentage of girls not meeting the EAR fell by 48 %. Intakes of vitamin D, Mg, Zn, Ca and K were also increased in this scenario. Those not meeting the EAR decreased for vitamin D, Zn and Mg in this model. The largest improvement in the percentage not meeting the EAR was observed for Ca in the dairy model. K intake was marginally improved, while Na intake increased but did not meaningfully impact the proportion of the population that had intakes above the AI *v*. baseline.

## Discussion

Our modelling exercises compared the effect of doubling plant-based foods *v*. the effect of doubling dairy foods on nutritional adequacy in adolescent females. It was hypothesized that doubling foods normally consumed in these food categories would be easier to achieve than imposing a behaviour change by doubling foods that are not frequently consumed. Additionally, if nutrient adequacy improved using this approach, it would provide a relatively simple message to advocate for improved health in this vulnerable population.

Scenario 1 doubled the intake of plant-based foods (fruits, vegetables, total grains, legumes, nuts, seeds and soya), while eggs, meat, poultry, fish and dairy intakes were decreased. Grain-based foods comprised the largest food group consumed within the plant-based food category. Total fat and saturated fat intake levels dropped as expected, but there was a small increase in added sugars. Dietary fibre consumption increased by 30 %, but over 90 % of adolescent girls still had intakes below the AI. There was a significant improvement in folate intake as the percentage not meeting the EAR dropped by almost 60 %. Vitamin E intakes were also improved. However, in this scenario, the proportion of girls not meeting the EAR for protein, Ca, Zn and vitamin D increased, two of which have been identified as nutrients of public health concern^(^ ^19^ ^)^. Our estimates of usual intakes indicated that 76 % of the adolescent girls have Ca intakes below the EAR, but in this scenario Ca intakes worsened, resulting in 92 % of adolescent girls not meeting the EAR. Consuming adequate amounts of dietary Ca during childhood and adolescence is critical for reaching peak bone mass, which may be important in reducing the risk of fractures and osteoporosis later in life^(^ ^16^ ^)^. Another striking observation of the plant-based food model (scenario 1) was that protein intakes were inadequate for 14·5 % of the population. This could be a cause for concern as protein intake is required to support numerous physiological processes and growth^(^ ^31^ ^)^.

Since we anticipated that protein intake would decrease in scenario 1, the second model increased the intake of protein-rich foods. Surprisingly, only vitamin E levels were different from baseline in this model. This finding is likely due to the fact that adolescent girls currently have very low intakes of legumes, nuts, seeds and soya products such that even doubling their intake had no real effect on nutrient intakes. The present study shows that adolescent girls consume little protein-rich plant foods; therefore there may be difficulty in getting them to increase their consumption to an amount that will lead to meaningful changes in nutrient intakes. This highlights the importance of ensuring that dietary recommendations are realistic and consider the behaviour changes required to meet recommendations.

Doubling the currently consumed dairy intake (scenario 3) resulted in a meaningful increase in total protein intake, reducing the proportion of girls not meeting the EAR by 48 %. Dairy is a source of high-quality protein, containing many peptides and bioactive factors that may have specific effects on growth^(^ ^8^ ^)^, as well as antihypertensive and anti-inflammatory activity^(^ ^8^ ^,^ ^33^ ^)^. There has been some concern that increased protein intake increases urinary Ca excretion by increasing the total acid load from protein metabolism; however, more recent research indicates this perception is not accurate in healthy individuals^(^ ^37^ ^,^ ^38^ ^)^. Furthermore, recommended intakes of dietary Ca take into account the typical protein intake of the population and adjusting Ca intake on the basis of protein intake generally is not recommended for children and adolescents^(^ ^16^ ^)^.

Increasing dairy intake reduced the proportion of adolescent girls not meeting the EAR for Ca from 76 to 41 %. Studies in children and adolescents have demonstrated that supplemental dietary Ca intake from dairy foods contributes to increases in bone mineral density, particularly in children with low baseline Ca intakes^(^ ^39^ ^–^ ^41^ ^)^. Furthermore, it has been observed in a nationally representative sample of women that low milk intake during childhood and adolescence was associated with low bone mineral content or bone mineral density of the hip in adulthood^(^ ^42^ ^)^. While adequate Ca intake during adolescence is essential, and dairy products provide the greatest amount of Ca in the diet^(^ ^43^ ^)^, there has been concern about dietary Ca negatively impacting the absorption of Fe^(^ ^44^ ^)^. A multi-country cross-sectional study showed a significant inverse correlation for dietary Ca intake (predominantly from dairy foods) and Fe stores; however, the quantitative effect was relatively small^(^ ^45^ ^)^. Additionally, results from a young adult female cohort who consumed a glass of milk with breakfast, lunch and dinner for four consecutive days showed no difference in the amount of non-haem Fe absorbed^(^ ^46^ ^)^.

Nutrients other than Ca are also involved in skeletal growth, including Mg, Zn and vitamin D^(^ ^47^ ^)^. Results from our dietary modelling exercises indicate that increasing dairy intake increased the intake of these nutrients, resulting in fewer adolescent girls falling below the EAR. Very few foods have naturally occurring vitamin D^(^ ^36^ ^)^. Fortified foods are the primary source of vitamin D in the American diet, with 60 % of vitamin D provided by milk for children aged 2–18 years^(^ ^48^ ^)^. However, results from our dairy model indicate that 80 % of adolescent girls were still not meeting their EAR for vitamin D. Similarly, while K intakes increased with increased dairy consumption, 99 % of the adolescents were still consuming amounts below the AI. It has been noted that less than 2 % of Americans have K intakes at or above the AI for K^(^ ^35^ ^)^. Yet the importance of dietary K in the adolescent female population was recently highlighted in a study by Moore *et al*., who reported that higher K intakes were inversely associated with blood pressure change^(^ ^14^ ^)^. The percentage of girls falling below the EAR for Fe, a nutrient singled out as being deficient in the diets of teenage girls, was not meaningfully improved by any of the modelling scenarios with the exception of a small decrease in scenario 1 (Table 3).

Although doubling dairy foods increased the intake of many key nutrients, it also increased the intakes of energy and saturated fat. Increased energy intake is a concern due to the rise in childhood and adolescent obesity. However, systematic reviews of the available data from observational and randomized controlled studies on children and adolescents indicate either an inverse or neutral relationship between dairy product and/or Ca intake and body weight and/or body fat^(^ ^9^ ^,^ ^10^ ^,^ ^49^ ^)^. The increased saturated fat intake associated with increased dairy intake may be less of a concern than previously believed. Several recent systematic reviews^(^ ^47^ ^,^ ^50^ ^–^ ^52^ ^)^ and meta-analyses^(^ ^53^ ^,^ ^54^ ^)^ have found either no association or an inverse association between the intake of dairy foods and cardiovascular risk, regardless of milk fat levels. This is consistent with a recent review that found no clear association between higher intake of saturated fat and all-cause mortality, CHD, CHD mortality, ischaemic stroke or type 2 diabetes among apparently healthy adults^(^ ^55^ ^)^.

Strengths of the present study include the use of a large, nationally representative database for determining the nutritional impact of changing the consumption patterns of plant and dairy foods; the use of the Food Pattern Equivalent Database to develop the diet modelling scenarios; use of usual intake methodology to assess nutrient adequacy; and modelling dietary changes that would be relatively simple to advocate. Limitations of the study include the use of self-reported intake data, which in the case of adolescent girls has been indicated to be under-reported^(^ ^56^ ^)^. None of our dietary models looked at the effect of doubling the intake of only fruits and vegetables *v*. all plant-based foods. Additionally, because it is difficult to disaggregate the individual foods from mixed dishes in NHANES, our models only reflect changes made to individual foods as consumed; foods in mixed dishes were not doubled. Accordingly, some food groups were not exactly doubled because a portion of their consumption occurs in mixed dishes. Data from NHANES 2009–2012 show that the total energy contribution of mixed dishes for adolescent females aged 9–18 years (*n* 1163) is approximately 1770 (se 75) kJ/d (423 (se 18) kcal/d), which translates to 23 (se 0·87) % of all energy in their diet (data not shown). Accordingly, future research should assess the impact of mixed dishes on diet quality as well as how changes in the foods associated with each mixed dish differ after the scenarios presented herein.

## Conclusions

Overall, our diet modelling exercises in adolescent girls demonstrate that general non-specific recommendations to increase plant-based foods at the expense of all animal foods lead to some nutritional benefits but can also result in unintended consequences. Doubling plant-based foods lowered intakes of total fat and saturated fat while increasing the intakes of dietary fibre, folate and vitamin E, but also resulted in lowered intakes for protein, Ca and vitamin D. If adolescent females were to follow this dietary pattern, only two of the five nutrients of public health concern identified by the 2015 Dietary Guidelines for Americans for adolescent females improved (fibre and Fe), while the intakes of the other three (Ca, vitamin D and K) worsened. Increasing protein-rich plant foods had no real impact because legumes, nuts, seeds and soya products are consumed at very low levels in this population. Doubling dairy foods led to dairy intakes that met recommended levels and helped to increase the intakes of protein, Ca, vitamin D, Mg and Zn; nutrients that have been singled out as important for adequate growth and bone health during the adolescent years. Additionally, the dairy model also meaningfully improved intakes for three of the nutrients of public health concern (Ca, vitamin D and K). Our data reinforce that dietary intake needs to be assessed as a whole and that dietary recommendations should consider the feasibility of realistically implementing the suggested behaviour changes required to meet nutrient needs.
